# Intraocular lens dislocation and tube shunt in the posterior chamber: a case report

**DOI:** 10.1186/s12886-015-0046-7

**Published:** 2015-06-21

**Authors:** Javier Moreno-Montañés, Alvaro Velázquez-Villoria, Alfonso L. Sabater, Angel Salinas-Alamán

**Affiliations:** Department of Ophthalmology, Clínica Universidad de Navarra, Universidad de Navarra, Avda. Pio XII, 36-31008 Pamplona, Spain

**Keywords:** Neovascular glaucoma, Ahmed glaucoma valve, Intraocular lens dislocation, Posterior vitrectomy

## Abstract

**Background:**

To describe management of a case of intraocular lens (IOL) and capsular bag (CB) dislocation in an eye with an Ahmed glaucoma valve in the posterior chamber.

**Case presentation:**

A 75-year-old pseudophakic man with open-angle glaucoma and diabetic retinopathy developed neovascular glaucoma. After two intravitreous injections of bevacizumab and panretinal photocoagulation were administered, the new vessels regressed. However, goniosynechiae were observed over 360° of the angle. An Ahmed glaucoma valve model FP7 was implanted with the tube in the posterior chamber with adequate intraocular pressure control. Nineteen years after cataract surgery, when the IOL-CB complex became dislocated, they were sutured transclerally to the sulcus without Ahmed glaucoma valve modification. After a coughing episode, the vitreous pushed the IOL-CB complex forward and the tube was behind the IOL-CB complex. A 25-gauge posterior vitrectomy was performed, and the tube was returned to in front of the optic of the IOL using a forceps tip through a sclerotomy.

**Conclusion:**

This case suggested that management of IOL-CB dislocation can modify glaucoma shunt function. A complete pars plana vitrectomy may be required in order to reposition the dislocated IOL-CB complex in the presence of a posterior chamber drainage tube implant.

## Background

Late intraocular lens (IOL)-capsular bag (CB) dislocation is a rare complication after uneventful cataract surgery that results from progressive zonular dehiscence in cases with pseudoexfoliation, retinitis pigmentosa, high myopia, vitreoretinal surgery, connective tissue disorders, and zonular trauma [1–5]. Little is known about the effect of IOL-CB dislocation on glaucoma in eyes that underwent trabeculectomy or have glaucoma valve shunts. We present a case of IOL-CB subluxation in an eye in which an Ahmed Glaucoma Valve (New World Medical, Inc., Rancho Cucamonga, CA) was implanted in an elderly patient with angle-closure glaucoma secondary to neovascular glaucoma. The tube was in the posterior chamber, and repositioning of the IOL altered glaucoma management. To our knowledge, no previous cases of IOL-CB complex subluxation in an eye with a glaucoma drainage valve have been reported.

## Case presentation

**Fig. 1:**
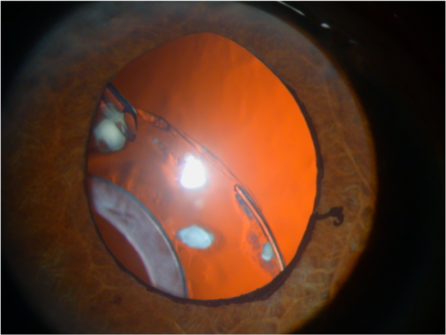
Inferior dislocation of the IOL-CB complex. The tube is positioned in front of the IOL-CB complex

**Fig. 2:**
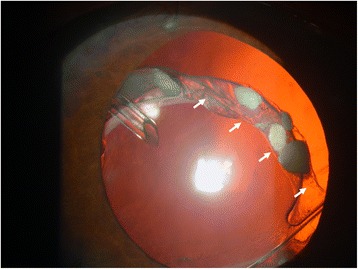
Suturing of the IOL-CB complex. The IOL-CB complex is sutured to the sulcus transsclerally but the superior CB is folded onto the optic IOL (arrows)

**Fig. 3:**
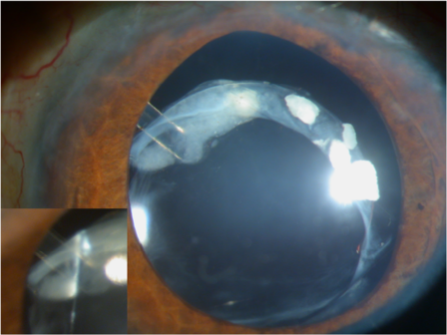
Subluxation of the IOL-CB complex. After an episode of coughing, the IOL-CB complex is pushed forward and the tube is placed behind the IOL

**Fig. 4:**
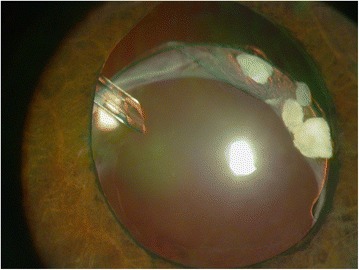
1 year postoperatively. One year after posterior vitrectomy, there are no changes or tube displacement behind the IOL

A 59-year-old man with diabetes underwent uneventful phacoemulsification in 1990 in the left eye with implantation of a one-piece polymethylmethacrylate IOL. Open-angle glaucoma developed postoperatively and was treated with brimonidine (Alphagan, Allergan, Irvine, CA), timolol maleate (Timoptic, Merck & Co, West Point, PA), and latanoprost ophthalmic solution (Xalatan, Pfizer Inc., New York). The right eye had phthisis bulbi secondary to a surgery to repair a retinal detachment. The patient did not instill sufficient medication and the optic disc damage increased. In 2006, iris rubeosis with uveal ectropion developed secondary to diabetic retinopathy. Gonioscopy found new vessels in the angle with synechiae in 360° of the angle. The intraocular pressure (IOP) was 45 mmHg in the left eye. The best-corrected visual acuity (BCVA) was 1 logarithm of the minimum angle of resolution (logMAR) unit in the left eye. Two intravitreous injections of bevacizumab (Avastin, Genentech Inc., South San Francisco, CA) (1.25 mg/0.05 mL) were administered in January and March 2007. Panretinal photocoagulation was applied at the same time to permanently ablate the ischemic retina, and the new vessels resolved. The IOP was 40 mmHg in the left eye after panretinal photocoagulation. In April 2007, an Ahmed Glaucoma Valve model FP7 was implanted with the plate positioned superonasally. Six months later, the bleb in the plate was encapsulated and the IOP was 14 mmHg. Two years later in April 2009, the IOL-CB complex became dislocated (Fig. 1). The BCVA was 2 logMAR units and the IOP was 16 mmHg. The patient denied any ocular or head trauma in the months before the examination. However, Valsalva maneuvers during an episode of coughing may have occurred because the patient had chronic obstructive lung disease. Transscleral 10/0 Prolene sutures were placed over and under both subluxated haptics through the anterior and posterior capsules to capture the haptics. The Prolene sutures were pulled to retract to the sclera and then were tied off. The IOL was centered, but the superior CB was folded over the optic IOL (Fig. 2). The BCVA was 1 logMAR unit and the IOP was 15 mmHg. Two months later, the BCVA decreased again after another episode of coughing, and the patient described intense ocular pain. The IOP was 40 mmHg and the BCVA was 2 logMAR units. The tube was placed behind the optic of the IOL; it was obstructed by the IOL, which increased the IOP (Fig. 3). A 25-gauge pars plana vitrectomy was performed, and the tube was again moved in front of the optic of the IOL using the tip of a forceps through one of the sutureless sclerotomies. One year after vitrectomy, the IOP was 15 mmHg and the BCVA was 1 logMAR unit. No other changes in the tube placement were seen (Fig. 4).

## Discussion

Intraocular injections of bevacizumab result in resolution of new vessels and panretinal photocoagulation causes ablation of the hypoxia in eyes with neovascular glaucoma. In cases with angle-closure glaucoma exceeding 330°, implantation of a drainage valve is recommended [6]. In the current case, 2 years after Ahmed tube implantation, the IOL-CB complex became dislocated, after which the IOL haptics were secured with transscleral Prolene sutures at the 3 and 9 o’clock positions using our previously described surgical technique [7]. Nevertheless, the superior CB was folded over the optic IOL (Fig. 2). No changes in shunt function and vitreous incarceration were seen in the tube. However, after an episode of coughing, the tube was placed behind the optic IOL. We hypothesized that during the Valsalva maneuver, the vitreous pushed the IOL forward and the optic IOL moved forward because only two stitches sutured the IOL haptics. This case suggested that management of IOL-CB complex dislocation can modify glaucoma shunt function.

Several surgical therapeutic options are available in cases of IOL-CB complex subluxation, including IOL repositioning or replacement [1, 2, 7–10]. The surgical approach depends on surgeon preferences and the degree of dislocation. The preferred approach for most surgeons is repositioning of the IOL-CB complex if it is not completely luxated into the vitreous, because surgical trauma to the corneal endothelium is reduced and the technique is performed easily [2, 7, 8]. A posterior pars plana vitrectomy is recommended when necessary, and in some cases it can be performed to remove vitreous strands from the haptics and capsular remnants, thus reducing the risk of peripheral retinal breaks [11]. In the current case, the IOL-CB complex was dislocated inferiorly without vitreous prolapse into the anterior chamber or vitreous strands, and the IOL-CB complex was repositioned without a posterior vitrectomy. However, the vitreous pushed the IOL forward and the tube was behind the IOL. The vitreous was removed completely during a 25-gauge sutureless posterior vitrectomy. In addition, the tube was repositioned in front of the optic using the tip of a forceps through one of the sutureless sclerotomies.

## Conclusions

We described a new complication after IOL-CB complex dislocation and sulcus transscleral suture in an eye with an Ahmed glaucoma implant. This case suggested that a posterior vitrectomy should be recommended as a one-stage surgery to suture the haptics transsclerally and the sulcus in cases with a tube in the posterior chamber. This combined procedure is especially necessary if the vitreous cavity is communicating with the anterior and posterior chambers, although the vitreous did not prolapse into the anterior chamber in the current case.

## Consent

A relative of the patient provided signed consent for publication of this case, which we can provide if requested.

The consent was obtained from the patients relative because the patient was died.
